# Preliminary development and psychometric evaluation of an unmet needs measure for adolescents and young adults with cancer: the Cancer Needs Questionnaire - Young People (CNQ-YP)

**DOI:** 10.1186/1477-7525-10-13

**Published:** 2012-01-30

**Authors:** Tara Clinton-McHarg, Mariko Carey, Rob Sanson-Fisher, Catherine D'Este, Anthony Shakeshaft

**Affiliations:** 1 Health Behaviour Research Group (HBRG), Priority Research Centre for Health Behaviour (PRCHB), University of Newcastle and the Hunter Medical Research Institute (HMRI), Callaghan, New South Wales, Australia; 2 Centre for Clinical Epidemiology and Biostatistics (CCEB), Priority Research Centre for Health Behaviour (PRCHB), University of Newcastle and the Hunter Medical Research Institute (HMRI), Callaghan, New South Wales, Australia; 3 National Drug and Alcohol Research Centre (NDARC), University of New South Wales, Sydney, New South Wales, Australia

**Keywords:** Adolescents and young adults, cancer, unmet needs, measure development, psychometric evaluation, reliability, validity

## Abstract

**Background:**

Adolescents and young adult (AYA) cancer survivors may have unique physical, psychological and social needs due to their cancer occurring at a critical phase of development. The aim of this study was to develop a psychometrically rigorous measure of unmet need to capture the specific needs of this group.

**Methods:**

Items were developed following a comprehensive literature review, focus groups with AYAs, and feedback from health care providers, researchers and other professionals. The measure was pilot tested with 32 AYA cancer survivors recruited through a state-based cancer registry to establish face and content validity. A main sample of 139 AYA cancer patients and survivors were recruited through seven treatment centres and invited to complete the questionnaire. To establish test-retest reliability, a sub-sample of 34 participants completed the measure a second time. Exploratory factor analysis was performed and the measure was assessed for internal consistency, discriminative validity, potential responsiveness and acceptability.

**Results:**

The Cancer Needs Questionnaire - Young People (CNQ-YP) has established face and content validity, and acceptability. The final measure has 70 items and six factors: Treatment Environment and Care (33 items); Feelings and Relationships (14 items); Daily Life (12 items); Information and Activities (5 items); Education (3 items); and Work (3 items). All domains achieved Cronbach's alpha values greater than 0.80. Item-to-item test-retest reliability was also high, with all but four items reaching weighted kappa values above 0.60.

**Conclusions:**

The CNQ-YP is the first multi-dimensional measure of unmet need which has been developed specifically for AYA cancer patients and survivors. The measure displays a strong factor structure, and excellent internal consistency and test-retest reliability. However, the small sample size has implications for the reliability of the statistical analyses undertaken, particularly the exploratory factor analysis. Future studies with a larger sample are recommended to confirm the factor structure of the measure. Longitudinal studies to establish responsiveness and predictive validity should also be undertaken.

## Background

Cancer in adolescents and young adults (AYAs) comprises approximately 0.5-2% of all cancer diagnoses [1-4]. While definitions of adolescence and young adulthood vary, AYAs are commonly defined as those aged 15 to 30 years [2,5,6]. Five year survival among AYAs varies depending on the type of cancer diagnosed, however, overall survival rates range between 73% and 83% [1,4,7]. Treatments may be lengthy and have been associated with both short-term side effects, including nausea (more than 35%)[8] and fatigue (30-93%)[9], and long-term consequences, such as infertility, secondary primary malignancies and cardiotoxicity [5].

Adolescence and young adulthood is a critical phase of physical, emotional, cognitive and social development [10,11]. As such, a cancer diagnosis may have profound effects on the lives of AYAs, interfering with the attainment of normal developmental milestones [11-13]. Increased dependence on family members at a time when AYAs would normally be developing their own independence may lead to changes in parent-child dynamics and conflict within the family [11]. Interruptions to schooling may result in subsequent difficulties with employment or educational achievements [5]. Body image concerns, difficulty accessing sexual health knowledge and obstacles to developing close interpersonal relationships may also serve as barriers for developing sexual identity in this group [12].

A first step in improving psychosocial outcomes for AYA cancer survivors is to accurately identify and prioritise their psychosocial needs [14,15]. A recent review of multi-dimensional measures of psychosocial health for AYA cancer survivors found two critical limitations. First, although most studies examined face and construct validity, few examined test-retest reliability or discriminative validity [16]. Second, there was an absence of validated unmet needs assessments, with most available measures focussing on quality of life [16]. Measures of unmet need are important as they provide direct information on consumer preferences that can be used to develop optimally relevant and cost-effective services [17].

Despite the lack of psychometrically rigorous unmet needs scales available for AYAs with cancer, recent studies in the United States have indicated that the number of unmet needs reported by this population group may be high [18]. As a consequence, there is a need to develop a scale to assess the unmet needs of AYAs which is relevant to both young people undergoing cancer treatment, as well as to those in the post-treatment survivorship phase.

The aim of this study was to establish the psychometric properties of the Cancer Needs Questionnaire -Young People (CNQ-YP), a measure designed to capture the perceived needs of AYA cancer patients and survivors. Specifically, the aims were to: 1) establish face and content validity; and 2) perform exploratory factor analysis, assess internal consistency, and examine the test-retest reliability, discriminative validity, responsiveness and acceptability of the measure.

## Methods

### Establish face and content validity

A number of steps were undertaken to develop the measure upon which psychometric evaluation was conducted. These are described below:

#### Literature review to identify items, domains and response scale

Medline, PsycINFO, Embase and CINAHL databases were searched to identify existing scales developed to assess the unmet needs of cancer patients or survivors of any age. The following keyword combinations: [neoplasm or cancer or oncol*] and [perceived need* or unmet need*] and [questionnaire or survey or measure or scale] were used in the database search. Publications reporting the development or psychometric properties of unmet needs measures were retained, and measures were examined to identify items and domains relevant to AYA cancer survivors.

The literature review identified 108 items, organised into seven domains, which were conceptually relevant to AYAs experiences of cancer. Relevant items were modified to reflect current life events among AYAs such as studying, early employment and supporting young families. Item readability was adapted to reflect the appropriate reading level of AYAs. An overall grade 6 reading level (confirmed by the Flesch-Kincaid Grade Level in Microsoft Word) was chosen because young people undergoing treatment for cancer may have missed substantial proportions of their schooling [11,19]. The response scale for the measure was adapted from the Cancer Needs Questionnaire (CNQ) and Supportive Care Needs Survey (SCNS). This response format has been rated as easy to follow and use by adult cancer populations [17,20,21].

#### Focus group with AYA cancer survivors

Adolescents and young adults with cancer were recruited through CanTeen Australia. CanTeen is the peak support organisation in Australia for AYAs aged 12-24 years affected by cancer. Six AYAs aged 14-19 years participated in the focus group. Using the draft questionnaire derived from the literature, participants were asked to list issues or needs which were most important or difficult for them and to provide other comments regarding item content, wording of questions and ease of completion.

#### Feedback from health professionals', researchers' and the general public

Health professionals from a regional hospital in New South Wales (NSW) Australia were nominated and invited by a paediatric oncologist to join a panel to review the draft needs instrument. Twelve health professionals participated in the panel which included one paediatric oncologist, two paediatric haematologists, three oncology nurses, one cancer care coordinator, one social worker, one psychologist, one oncology pharmacist, one physiotherapist and one occupational therapist. All health professionals had experience working directly with AYA cancer populations. The panel were asked to provide feedback on the content of the survey and suggest additional items.

Eight researchers from Australia and Canada with expertise in the development of unmet needs measures for cancer populations were asked to provide critical feedback on the domains, items and response scale of the measure via email. A convenience sample of twelve individuals from the general population, who were known to the research team and were not from a medical or research background, were also asked to provide feedback on the questionnaire in terms of its language and clarity. This sample included adult professionals, such as school teachers and engineers, as well as parents and AYAs who had not experienced cancer.

#### Revision of measure

Feedback and advice received from all participants led to a revised response scale and stem for the measure, and one extra domain (total of eight domains). The initial number of items was also modified. Four items were removed, and 31 items added taking the total number of items from 108 to 139.

#### Pilot testing

The measure was pilot tested with AYAs recruited from a population-based cancer registry in Australia. Adolescents and young adults who participated in the pilot were: 1) aged 14 to 19 years at diagnosis; and 2) had been diagnosed with cancer in the last five years (between January 1 2002 and December 31 2007). The upper age limit of 19 years at diagnosis was selected, as the World Health Organisation (WHO) defines adolescents as being between 10-19 years of age [22]. The lower limit was chosen because, in Australia, AYAs aged 14 years and older have the legal right to make their own decisions about the type of health care they receive [23]. The eligibility criteria of 5 years since diagnosis ensured that the sample comprised of young people up to the age of 24 years. Details of the pilot study are presented elsewhere [24].

Of the 32 AYAs who participated in the pilot test, 53% were female and 90% had completed treatment. A quarter of participants (25%, n = 8) commented that they found the response time-frame, "in the past month", did not allow them to express all the concerns about unmet needs they had experienced throughout their cancer journey, especially during treatment. Therefore, the time-frame of the response scale was modified to "any time since your cancer diagnosis" for five domains (Cancer Treatment Staff, Cancer Treatment Centre, Education, Work and Information). For the remaining three domains (Feelings, Relationships and Daily Life), the response time-frame remained "in the last month".

### Establish psychometric properties

#### Description of the Cancer Needs Questionnaire - Young People (CNQ-YP)

Face and content validity was established using the procedures above. The resulting measure had 139 items presented in eight domains: 1) Cancer Treatment Staff (36 items); 2) Cancer Treatment Centre (11 items); 3) Education (10 items); 4) Work (10 items); 5) Information (9 items); 6) Feelings (35 items); 7) Relationships (18 items); and 8) Daily Life (10 items). Items were rated using a five-point response scale from "No Need" to "Very High Need".

Items in the Education, Work and Relationships domain were only answered if they were relevant to the AYA's situation. Two screening questions determined whether the young person was currently studying, two screening questions determined whether the young person was currently employed, and one screening question determined if the AYA had a "spouse/partner or boyfriend/girlfriend" or "sibling/s or step-brothers/sisters".

#### Participants

Adolescents and young adults were eligible to participate in the main trial if they: 1) had received treatment for cancer at one of seven identified treatment centres in Australia; 2) had been diagnosed with an invasive cancer in the last five years (between 1 January 2004 and 31 December 2009); 3) were aged 14 to 25 years inclusive at the time of diagnosis (e.g. currently up to 30 years of age); and 4) had not participated in the pilot study. Participants were also confirmed by their treating clinician as: 5) having a life expectancy of at least 12 months; 6) being physically and mentally able to complete a survey; and 7) being sufficiently literate in English to complete the measure.

#### Procedure

Relevant institutional ethics approvals were obtained for the study. Recruitment commenced in August 2009 and data collection was completed in May 2010. Adolescents and young adults who met the eligibility criteria were identified from medical records at each treatment centre. The principal clinician at each treatment centre mailed a study information pack to eligible AYAs and sought permission to release their contact details to the research team. Those who agreed were mailed a survey packet containing a reply paid envelope, a copy of the CNQ-YP, and questions about demographic characteristics including: marital status; language preferences; living arrangements; education; preferences regarding the survey format and locations for survey completion; and feedback of results to health professionals, treatment centres, researchers and other organisations. Reminder letters were sent to non-responders at two weeks and a reminder phone call was made at four weeks following the initial mailout.

Return of the completed measure was taken as a participant's consent for their data to be included in the study. In order to evaluate the test-retest reliability of the measure, all participants who agreed to be contacted again were sent copies of the CNQ-YP one week following receipt of their initial survey and asked to complete the measure a second time. The one-week time-frame was chosen to minimise the chance that patients had substantial changes in their unmet needs or could recall their previous responses [25].

#### Statistical analysis

The demographic characteristics of AYAs were reported using descriptive statistics (frequencies, proportions, means, medians and 95% confidence intervals). Differences in the characteristics of consenters and non-consenters were examined using the Chi-square statistic.

### Psychometric evaluation

#### Factor analysis

Items which had greater than 90% of respondents indicating the same level of need and items answered by ≤ 10% of respondents were excluded from the measure. Observations with a missing value for any of the included items were excluded from the factor analysis using list-wise deletion. Exploratory factor analysis using the principal components method (PCA) and Eigenvalue > 1 rule was performed. Factors were orthogonally rotated using the varimax procedure and factors which accounted for greater than 5% of the variance were considered important. Items which had a factor loading of > 0.40 on only one factor, or where > 20% of participants indicated having a high or very high need for the item, were kept [26]. Spearman's rank correlation coefficient was then used to identify items which were highly correlated with each other (correlations > 0.90) and had < 20% of participants indicating a high or very high need, which could be further excluded from the factors.

#### Internal consistency

Items which had an item-total correlation with the total scale of < 0.20 were discarded from the measure. For each factor, a Cronbach's coefficient alpha (α) value of > 0.70 and < 0.95 was considered acceptable.

#### Test-retest reliability

A weighted Cohen's kappa coefficient (κ), was used to measure the level of agreement between responses at baseline (time 1) and retest (time 2). Items which had a weighted kappa of > 0.60 were considered to have excellent test-retest reliability and were retained. To ensure items related to high unmet needs were not dismissed, items which did not obtain a weighted kappa of > 0.60 but for which > 20% of participants indicated having a high or very high need, were also kept.

#### Discriminative validity

In order to compare different groups of participants, factor scores were calculated by summing all raw scores for items within the factor and dividing by the number of non-missing items. As all items in the measure were worded and scored in the same positive direction, no reversing of response scores for items was required. Observations with missing values for > 50% of items within a factor were excluded from the analysis.

Based on previous research [27-30], it was hypothesised that young people receiving treatment would have a higher median factor score for all factors, compared with young people who had finished treatment. A non-parametric Wilcoxon rank-sum test was computed to determine if any significant differences between the factor scores of these two groups existed.

#### Responsiveness

Responsiveness was gauged using floor and ceiling effects. Factors where less than 5% of participants scored the lowest possible score or the highest possible score were considered acceptable.

#### Acceptability

Acceptability of the measure was assessed using the following four questions: 1) "I found the instructions easy to follow"; 2) "I found the questions clear"; 3) "I found the answer choices easy to understand"; and 4) "I found the questions distressing". Items were scored on a five point Likert scale from "Strongly Disagree" to "Strongly Agree". The acceptability of the measure was reported using frequencies, proportions, and 95% confidence intervals.

## Results

### Participants, response rates and consent bias

Five hundred and seventy-seven eligible AYAs were identified by their clinicians' at the treatment centres, and 280 of these (49%) consented to be contacted by the research team. Of the 280 young people contacted, 139 (50%) returned a questionnaire. Of the 139 participants who completed the measure at baseline (time 1), 101 (73%) consented to be contacted a second time and were sent a retest survey, with 34 (34%) completing the measure at time 2. The time between being sent and returning the retest measure ranged between 9 and 64 days, with a median of 24 days (Q1 = 16 days, Q3 = 30 days).

The demographic characteristics of AYAs who consented to participate in the baseline study, and those AYAs who did not consent, can be seen in Table 1. Consenters were significantly younger than non-consenters, and females were over-represented in the consenting sample. There were no significant differences between the demographic characteristics of AYAs who completed both the baseline and retest surveys, compared to AYAs who completed the baseline survey only.

**Table 1:** Demographic characteristics of consenters and non-consenters for the baseline study

Demographic Characteristic	Non-consenters (n = 438)	Consenters (n = 139)	Test statistic		
	**n (%)**	**n (%)**	***χ***^**2**^	**df**	***p***
	
Female	188 (43)	89 (64)	18.8	1	< 0.01
Non-haematological	243 (56)	73 (53)	0.31	1	0.58
≥ 2 years post-diagnosis	293 (68)	99 (71)	0.56	1	0.45
	
	**Median (Q1-Q3)**	**Median (Q1-Q3)**	**z**		***p***
	
Age at diagnosis	22 (19-24)	21 (18-23)	2.24		0.03

### Psychometric evaluation

#### Factor analysis

No items had > 90% of participants reporting the same level of need, indicating reasonable variability of responses within items. Only two items in the measure had missing values greater than 10% (items 126 and 128 from the Relationships domain). These two items were removed from the measure, leaving 137 items prior to conducting factor analysis.

Of the 139 participants who completed the measure, 111 observations had no missing values for any items and were included in the analysis. Due to the screening questions, only a subset of AYAs completed the domains related to Education and Work, meaning there were different numbers of observations relating to different participants. Therefore, the initial factor analysis was undertaken without these two domains. Following the initial factor analysis, the Work and Education domains were added to the analysis, one at a time.

Initial factor analysis of the six main domains (112 of 137 items) revealed 18 factors with Eigenvalues > 1. When the factors were orthogonally rotated, three factors accounted for greater than 5% of the variance. A three-factor forced analysis and rotation confirmed that the three-factor structure was the simplest and clearest. Ninety-five items fell into these three factors and accounted for 58% of the total variance, 17 items were removed. When the 20 items from the Education and Work domains were independently added to the factor analysis, two additional factors were identified and 10 items were removed, taking the total number of factors to five. Five additional items from the Relationships sub-domains (Partners and Siblings) had significant loadings > 0.40 on a single factor and all were added to Factor 2.

An item-item Spearman correlation matrix for each of the five factors and 108 items revealed that two items from Factor 2 had correlations > 0.90. However, these items appeared to capture different aspects of the patient/staff relationship; therefore, neither item was removed from the measure.

#### Internal consistency

Item-total correlations for items within all five factors were > 0.20 and ranged from 0.33 to 0.88. All factors had Cronbach's alphas of > 0.70, indicating good internal consistency.

#### Test-retest reliability

Although traditionally performed prior to factor analysis, test-retest reliability was performed after factor analysis in the present study due to the small sample size (n = 34). This allowed the number of items included in the test-retest analysis to be reduced, thereby limiting the likelihood of type 1 error. Weighted kappa values between responses at time 1 and time 2 ranged from 0.09 to 0.94. Twenty-four items had a weighted kappa of < 0.60 and < 20% of participants indicating a high or very high need, and were therefore excluded from the measure leaving 84 items.

#### Revised factor analysis and internal consistency

Following test-retest reliability analysis, factor analysis was repeated to confirm the factor structure of the measure. One hundred and sixteen observations had no missing values for any items and were included in the analysis. The number of important factors increased from five to six. The four main factors explained 63% of the variance. Fourteen items did not have unique factor loadings > 0.40 and were removed from the measure, leaving 70 items. All but six items loaded on the same factor as in the previous factor analysis, with five of these loading on the new factor. The items and factor loadings corresponding to these six factors are presented in Table 2.

**Table 2:** Factor structure of the CNQ-YP from the revised factor analysis

Item number		Description of item	Factor loading					
			
			Factor 1	Factor 2	Factor 3	Factor 4	Factor 5	Factor 6
**Factor 1 - Treatment Environment and Care (n = 116)**								

Cancer treatment staff telling me:	1	about my diagnosis	0.76					
	2	what might happen during treatment	0.77					
	4	whether I had the option to decline treatment	0.54					
	5	about the short-term side-effects of treatment	0.73					
	6	about the long-term side-effects of treatment	0.65					
	7	my chances of a full recovery	0.78					
	8	what would happen when treatment finished	0.71					
	9	whether I would be able to have children	0.60					
	12	whether my treatment was working	0.87					
	13	my test results as soon as possible	0.87					
	14	the way I felt was normal	0.80					
	15	how to manage my medication	0.82					
	16	what I could do to stay healthy	0.67					
	17	what to do if I noticed a particular side-effect	0.74					
Having cancer treatment staff who:	20	listened to my concerns	0.89					
	21	treated me as an individual	0.87					
	22	were respectful	0.93					
	23	were approachable	0.91					
	24	were friendly	0.91					
	25	could have a laugh with me	0.90					
	26	explained what they were doing	0.89					
	27	spoke to me in a way that I could understand	0.90					
	28	let me talk about my feelings	0.77					
	29	let me ask questions	0.90					
	30	let me make decisions about my treatment	0.74					
	31	talked to me in private, without my family	0.66					
Being able to have:	39	time to myself	0.55					
	41	privacy	0.51					
	42	pleasant surroundings	0.52					
	43	good food	0.45					
	46	a choice of cancer specialists	0.64					
	47	the same cancer staff throughout treatment	0.63					
	48	a choice of times for appointments	0.64					

**Factor 2 - Daily Life (n = 116)**								

Being able to:	110	make plans or think about the future		0.51				
Coping with:	102	changes in my physical ability		0.68				
	103	changes in my appearance		0.69				
	107	not being able to do the same things as other people my age		0.75				
	117	my parent/s being over-protective		0.49				
Managing:	134	pain		0.65				
	135	medication		0.48				
	136	physical side-effects of treatment		0.76				
	137	feeling tired		0.72				
	138	loss of mobility		0.65				
	142	to take part in social activities		0.71				
	143	to travel to social events		0.67				

**Factor 3 - Feelings and Relationships (n = 116)**								

Feeling:	84	frustrated			0.56			
	86	anxious or nervous			0.73			
Worrying about:	91	my cancer spreading			0.76			
	92	my cancer returning			0.66			
	93	whether my cancer treatment has worked			0.68			
	95	having cancer treatment			0.70			
	97	how my family is coping			0.68			
Finding:	98	inner strength			0.66			
Being able to:	112	accept my diagnosis			0.60			
	113	be independent			0.51			
Coping with:	125	changes in my relationship with my partner			0.53*			
	131	changes in my relationships with my sibling/s			0.59*			
Knowing how to:	132	ask my sibling/s for support			0.51*			
	133	give support to my sibling/s			0.52*			

**Factor 4 - Information and Activities (n = 116)**								

Being able to:	37	spend time with people my own age				0.44		
	38	talk to people my age who had been through a similar experience				0.69		
Being able to have:	44	leisure spaces and activities				0.45		
Finding information that:	75	was specifically designed for me				0.61		
	81	described relaxation techniques				0.54		

**Factor 5 - Education (n = 65)**								

Being able to:	56	attend classes					0.69	
	58	get extensions or special consideration					0.74	
	59	get guidance about study options or future career paths					0.56	

**Factor 6 - Work (n = 90)**								

Knowing:	71	how much work I would miss						0.67
	72	how to ask managers/co-workers for support						0.78
	74	that managers/co-workers had support to help them cope						0.76

**% of Total Variance**			**31%**	**13%**	**11%**	**8%**	**n/a**	**n/a**

*AYAs only completed these items if relevant to their situation. Item 125 was completed by n = 54 participants, Items 131, 132 and 133 were completed by n = 96 participants. These items do not form part of the total variance explained.

Internal consistency of the measure was re-calculated on the shortened measure. Item-total correlations of the 70 items for all six factors were still > 0.20 and ranged from 0.32 to 0.90. All factors maintained alphas > 0.70 (Table 3).

**Table 3:** Cronbach's alpha for each Factor of the CNQ-YP

Description of factor	Number of items	Cronbach's alpha
Factor 1 - Treatment Environment/Care	33	0.98
Factor 2 - Daily Life	12	0.94
Factor 3 - Feelings/Relationships	14	0.92
Factor 4 - Information/Activities	5	0.83
Factor 5 - Education	3	0.82
Factor 6 - Work	3	0.89
**Total Scale**	**70**	**0.98**

#### Discriminative validity

Of the 139 AYAs who completed the measure, six participants were unsure of their treatment status and were excluded from the known-groups comparison. Participants receiving treatment had higher median factor scores than those who had finished treatment for all factors except Factors 5 and 6 ("Education and Work"), however these differences were not statistically significant (see Table 4).

**Table 4:** Comparison of factor scores between AYAs receiving treatment and AYAs finished treatment

Factor	Receiving Treatment				Finished Treatment				Wilcoxon rank sum			
	
	n	median	Q1	Q3	n	median	Q1	Q3	*z*	*p*		
1 - Treatment Environment/Care	17	1.8	1.1	2.1	116	1.5	1.2	2.2	0.32	0.75		
2 - Daily Life	17	2.3	1.1	3.2	115	1.4	1.0	2.2	1.46	0.14		
3 - Feelings/Relationships	17	2.4	1.6	2.6	115	1.5	1.2	2.3	1.81	0.07		
4 - Information/Activities	17	3.6	1.6	3.8	116	2.2	1.5	2.8	1.58	0.11		
5 - Education	11	1.3	1.0	2.0	67	1.7	1.0	2.7	-0.92	0.36		
6 - Work	13	1.3	1.0	3.0	94	1.3	1.0	2.0	0.53	0.60		

#### Potential responsiveness

The proportion of participants who scored the minimum and maximum scores for each factor can be seen in Table 5. The proportion of participants ranged from 0% to 5.1% for the maximum score to 8.3% to 43% for the minimum score, with large proportions of participants having floor effects in the "Education" and "Work" factors (42% and 43% respectively).

**Table 5:** Floor and ceiling effects per factor

Factor		Lowest possible score	Highest possible score
	
	n	n (%)	n (%)
Factor 1 - Treatment Environment/Care	133	11 (8.3)	1 (0.8)
Factor 2 - Daily Life	132	36 (27)	0 (0.0)
Factor 3 - Feelings/Relationships	132	19 (14)	0 (0.0)
Factor 4 - Information/Activities	133	16 (12)	6 (4.5)
Factor 5 - Education	78	33 (42)	4 (5.1)
Factor 6 - Work	107	46 (43)	4 (3.7)

#### Acceptability

When asked about the acceptability of the measure, 80% of participants agreed that the instructions were easy to follow (n = 111, CI 72-86%), 73% agreed that the questions were clear (n = 102, CI 65-80%) and 56% (n = 78, CI 48-64%) agreed that the answer choices were easy to understand. Seventy-eight percent (n = 108, CI 71-84%) of AYAs disagreed that the questions were distressing.

## Discussion

This research attempted to establish the face and content validity, factor structure, internal consistency, test-retest reliability, discriminative validity, potential responsiveness and acceptability of the CNQ-YP using rigorous psychometric criteria. Factor analysis and the assessment of test-retest reliability resulted in a final measure with six factors and 70 items (75 items including the five screening items): 1) Treatment Environment and Care (33 items); 2) Daily Life (12 items); 3) Feelings and Relationships (15 items including 1 screening item); 4) Information and Activities (5 items); 5) Education (5 items including 2 screening items); and 6) Work (5 items including 2 screening items).

There are a number of limitations related to the study sample and methodology which should be considered when interpreting these results.

### Limitations

The primary limitation of the research was the size of the sample achieved. Only 139 AYAs recruited through the seven treatment centres completed and returned the measure (an overall response rate of 50%). Other studies describing the development of measures for AYA cancer patients have reported response rates of around 90% [28,30,31]. However, the age range of these samples (8-20 years) was lower than in the current study (16-30 years). One study describing the development of a measure for a similar age group (16-28 years) only achieved a response rate of 53% [32]. Reasons for lower response rates with older AYA samples, compared with younger samples, are speculative. AYAs in this age group are highly mobile [7,33]. Therefore, it is possible that a large proportion of AYAs may not have received the questionnaire because of incorrect contact details. It is also possible that some young people were not interested in participating in this type of research or perceived that the research was not relevant to their current circumstances. Low participation may be especially applicable to psychosocial research studies where personal issues such as feelings and emotions related to cancer are discussed. Similar results have been found with adult breast cancer survivors [34], indicating that participants in psychosocial cancer research may be self-selected and are only representative of a sub-population of survivors who wish to talk about their experiences.

The small sample size has implications for the statistical analyses undertaken, particularly the exploratory factor analysis. When performing factor analysis it is recommended that the number of participants in the sample be at least five times the number of items in the measure [35]. As there were 139 items in the original measure, only a 1:1 item-to-participant ratio was actually achieved. This may have meant that some important items reported as high or very high unmet needs by smaller sub-samples of participants did not achieve high correlations with other items or factors in the measure [36]. However, as the inclusion criteria allowed items which had greater than 20% of participants reporting a high or very high level of need to be retained, it is unlikely that items considered important by sub-samples of AYAs were excluded.

The sample for the test-retest analysis was also small and the median time to return the retest measure was greater than the recommended 2-14 days [37]. Consequently, responses to the retest measure may have reflected a change in participants' needs [37]. Despite the longer than recommended retest period, the majority of items had acceptable kappa values (> 0.60), and as the time-frame for the response scale was either "any time since your cancer diagnosis" or "in the last month", it is unlikely that the longer period of retest would have greatly affected the overall responses obtained. The inclusion criteria also allowed items which had a low weighted kappa value but a large proportion of participants (> 20%) reporting a high or very high level of need to remain in the measure, further ensuring that important items were not excluded.

Although small, the overall sample included participants from five states and both large- and small-volume treatment centres for AYA cancer patients. Therefore, it is likely that a wide range of young people were involved. Those who consented to take part in the study were slightly younger and more likely to be female than those who did not consent. However, participation by AYAs with a range of cancer types, at different stages since their cancer diagnoses, increases the probability that the items identified as important in the measure represent the views of the larger AYA cancer population.

Finally it should be noted that, apart from infertility, issues surrounding other aspects of sexual health and intimacy were not raised in focus group discussions or in the pilot study. Such items were therefore not incorporated into the measure. The absence of these items may reflect the young age of participants in the pilot study compared to participants in the main trial. It is recommended that issues surrounding sexual health are further investigated in future scale development.

### Psychometric strengths of the CNQ-YP

Despite the difficulties with recruitment, the current study had a number of strengths related to the psychometric development of the CNQ-YP and the measure compared favourably with recognised psychometric criteria. Reliability and validity of the CNQ-YP was examined using appropriate psychometric methods. The final factor structure of the CNQ-YP showed that the four main factors accounted for 63% of the variance. This was considered acceptable, as the average variance accounted for by exploratory factor analysis is around 60% [36]. This outcome also compares well with other quality of life measures developed for AYA cancer survivors, such as the Adolescent Quality of Life Instrument (AQoL) which reported having six factors representing 67% of the variance [31], and the Quality of Life - Cancer Survivors (QOL-CS) instrument which also has six factors accounting for 56% of the variance [32].

In addition, the CNQ-YP achieved high Cronbach's alphas, with all six factors reporting alphas greater than 0.80. It is possible that some alpha values may have been artificially high due to the large number of items in the factors [25,38]. However, no items had correlations < 0.20, and alphas > 0.80 were also reached in Factors 5 and 6 (Work and Education), both of which had only three items. Although most of the available quality of life measures developed for AYA cancer survivors have at least some domains with alphas > 0.70, no scales achieved alphas greater than 0.70 for all domains [16]. In the case of the Perceived Illness Experience Scale (PIE), only two out of nine domains had alpha values > 0.70, showing variability in the internal consistency of these measures [16].

A further strength of this study is that it assessed test-retest reliability, unlike many other studies reporting the development of measures for cancer patients and survivors [16,39]. All but four items in the measure had weighted kappa values > 0.60, and these four items all had weighted kappas > 0.49. In comparison, a recent review found that test-retest reliability was reported for only one instrument measuring quality of life in AYA cancer survivors [16]. However, for this measure ICCs were only reported at the domain level rather than the item level. This can be misleading as, although total agreement levels for the domain may be high, agreement for individual items may vary [40].

### Recommended improvements for the CNQ-YP

In the current study, the CNQ-YP was unable to distinguish between AYAs currently receiving treatment and those who had completed treatment. For Factors 1, 4, 5 and 6 (Treatment Environment and Care, Information and Activities, Education, and Work) this may have been because the response time-frame was "any time since diagnosis". Therefore, patients who had completed treatment may have been reflecting a need level they had while receiving treatment. The small sample size in the receiving treatment group (n = 17) may have also limited the power for hypothesis testing. There was a non-significant trend toward AYAs receiving treatment reporting higher needs related to Feelings and Relationships. This could be further explored with a larger sample.

The CNQ-YP did not appear to have a ceiling effect (< 5.1% of participants scored the highest possible score in each domain). However, there was a large floor effect for all domains. This may have implications for intervention studies where researchers wish to measure a reduction in needs, as a large proportion of participants (between 8.3% and 43%) are already scoring the minimum possible scores for each factor. However, factors with the largest floor effects (Education 42% and Work 43%) were only completed by a sub-group of participants. Therefore, a larger sample of AYAs may produce different results. These floor effects may also indicate that the majority of young people do not experience high levels of need in these areas. However, the Education factor also had the highest ceiling effect (5.1%), suggesting that this is probably not the case.

Testing of convergent and divergent validity, responsiveness and predictive validity was beyond the scope of the present study. It is recommended that future psychometric testing of the measure be undertaken to explore these issues further. The small sample size means that the factor structure achieved in the current study may not be reproducible. Therefore, it is also recommended that confirmatory factor analysis with a larger sample of AYA cancer survivors be conducted prior to using the measure in clinical practice [36,41].

## Conclusions

The CNQ-YP is the first multi-dimensional measure of unmet need which has been developed specifically for AYA cancer patients and survivors. The measure displays a strong factor structure, and good internal consistency and test-retest reliability. Future studies with a larger sample size are recommended to determine the discriminate validity and floor and ceiling effects of the measure. Longitudinal studies to establish responsiveness and predictive validity should also be undertaken.

## Competing interests

The authors declare that they have no competing interests.

## Authors' contributions

TCM, MC, RSF and AS were responsible for the initial study design. All authors developed and refined the measure. TCM and MC coordinated recruitment and data collection. TCM, MC and CD conducted psychometric analysis. All authors contributed to drafting, revising and approving the final manuscript.
